# Biological effects of *trans, trans*-farnesol in *Leishmania amazonensis*

**DOI:** 10.3389/fcimb.2023.1221246

**Published:** 2023-11-16

**Authors:** Liliane Sena Pinheiro, Valter Viana Andrade-Neto, Marcio Mantuano-Barradas, Elisa Cavalcante Pereira, Rodrigo Cesar Fernandes Barbosa, Marcia Cristina Campos de Oliveira, Rubem Figueiredo Sadok Menna-Barreto, Edézio Ferreira Cunha-Júnior, Eduardo Caio Torres-Santos

**Affiliations:** ^1^Laboratório de Bioquímica de Tripanosomatídeos, Instituto Oswaldo Cruz, Fundação Oswaldo Cruz (FIOCRUZ), Rio de Janeiro, RJ, Brazil; ^2^Universidade Federal dos Vales do Jequitinhonha e Mucuri, Teófilo Otoni, MG, Brazil; ^3^Instituto de Química, Universidade Federal Rural do Rio de Janeiro, Seropédica, RJ, Brazil; ^4^Laboratório de Biologia Celular, Instituto Oswaldo Cruz, Fundação Oswaldo Cruz (FIOCRUZ), Rio de Janeiro, RJ, Brazil; ^5^Laboratório de Imunoparasitologia, Unidade Integrada de Pesquisa em Produtos Bioativos e Biociências, Centro Multidisciplinar UFRJ-Macaé, Universidade Federal do Rio de Janeiro, Macaé, Brazil

**Keywords:** *Leishmania*, farnesol, farnesene, isoprenoid, sesquiterpenoid, lipid secretome

## Abstract

**Introduction:**

Farnesol, derived from farnesyl pyrophosphate in the sterols biosynthetic pathway, is a molecule with three unsaturations and four possible isomers. *Candida albicans* predominantly secretes the *trans*, *trans*-farnesol (*t*, *t*-FOH) isomer, known for its role in regulating the virulence of various fungi species and modulating morphological transition processes. Notably, the evolutionary divergence in sterol biosynthesis between fungi, including *Candida albicans*, and trypanosomatids resulted in the synthesis of sterols with the ergostane skeleton, distinct from cholesterol. This study aims to assess the impact of exogenously added *trans*, *trans*-farnesol on the proliferative ability of *Leishmania amazonensis* and to identify its presence in the lipid secretome of the parasite.

**Methods:**

The study involved the addition of exogenous *trans*, *trans*-farnesol to evaluate its interference with the proliferation of *L. amazonensis* promastigotes. Proliferation, cell cycle, DNA fragmentation, and mitochondrial functionality were assessed as indicators of the effects of *trans*, *trans*-farnesol. Additionally, lipid secretome analysis was conducted, focusing on the detection of *trans*, *trans*-farnesol and related products derived from the precursor, farnesyl pyrophosphate. *In silico* analysis was employed to identify the sequence for the farnesene synthase gene responsible for producing these isoprenoids in the *Leishmania* genome.

**Results:**

Exogenously added *trans*, *trans*-farnesol was found to interfere with the proliferation of *L. amazonensis* promastigotes, inhibiting the cell cycle without causing DNA fragmentation or loss of mitochondrial functionality. Despite the absence of *trans*, *trans*-farnesol in the culture supernatant, other products derived from farnesyl pyrophosphate, specifically α-farnesene and β-farnesene, were detected starting on the fourth day of culture, continuing to increase until the tenth day. Furthermore, the identification of the farnesene synthase gene in the *Leishmania* genome through in silico analysis provided insights into the enzymatic basis of isoprenoid production.

**Discussion:**

The findings collectively offer the first insights into the mechanism of action of farnesol on *L. amazonensis*. While *trans*, *trans*-farnesol was not detected in the lipid secretome, the presence of α-farnesene and β-farnesene suggests alternative pathways or modifications in the isoprenoid metabolism of the parasite. The inhibitory effects on proliferation and cell cycle without inducing DNA fragmentation or mitochondrial dysfunction raise questions about the specific targets and pathways affected by exogenous *trans*, *trans*-farnesol. The identification of the farnesene synthase gene provides a molecular basis for understanding the synthesis of related isoprenoids in *Leishmania*. Further exploration of these mechanisms may contribute to the development of novel therapeutic strategies against *Leishmania* infections.

## Introduction

The mevalonate pathway is a biosynthetic route that originates cholesterol in mammals and ergosterol in fungi and trypanosomes. (Goldstein and Brown, 1990; Roberts et al., 2003; Nickerson et al., 2006). This pathway also generates precursors of essential isoprenoids, including farnesyl pyrophosphate (FPP), which serves as the substrate for the synthesis of farnesol (FOH), α-farnesene, and β-farnesene through dephosphorylation These metabolites play crucial roles in cell signaling processes that are essential for survival, growth, differentiation, and proliferation of eukaryotic cells. Moreover, certain enzymes involved in isoprenoid metabolism were identified in the pheromone gland of *Lutzomyia longipalpis*, the primary vector of the protozoan parasite *Leishmania infantum* in Latin America (González-Caballero et al., 2013; Spiegel et al., 2016). Both forms of farnesene are odorant components of several plants, included some used in human alimentation, such as chamomile (Rafieiolhossaini et al., 2012). Furthermore, β-farnesene is well known as an aphid alarm pheromone (Bowers et al., 1972; Vandermoten et al., 2012), and a study has shown that it is a feeding stimulant for *Lutzomyia longipalpis* (Tesh et al., 1992).

Besides the metabolic importance of FOH within the cells, it has been shown that in particular concentrations in microenvironment, FOH inhibits cell proliferation and induces apoptosis in a broad range of cell types (Semighini et al., 2008; Joo and Jetten, 2010).

It has been demonstrated that *Candida albicans*, a medically significant polymorphic fungus, synthesizes and secretes FOH. Through *quorum sensing*-type signaling, this isoprenoid modulates morphological transition processes and regulates the fungus’s virulence. While FOH can exist in four isomeric forms, only the *trans, trans*- FOH (*t*, *t*- FOH) has been implicated in signaling activity within *C. albicans* cultures (Hornby et al., 2001). Furthermore, studies indicate that FOH’s effect may vary depending on the culture conditions. Therefore, in richer nutrient medium, the fungus has a proportionally greater ability to tolerate higher concentrations of FOH. In this context, *t, t*- FOH can exhibit a toxic effect on the fungi, inducing apoptosis, or it can act as a signaling molecule (Langford et al., 2010).

Trypanosomatids and fungi have their own sterols synthesis machinery that differs in some steps from the mammalian pathway. Due to evolutionary divergence, fungi and trypanosomatids do not synthesize cholesterol; instead, their sterols have an ergostane skeleton (Roberts et al., 2003). However, it is still unknown which evolutionary pressures led fungi and trypanosomatids to differentiate their sterol metabolism from that other eukaryotes, especially considering their phylogenetic distance. Thanks to the discovery of this biochemical convergence, the activity of clinically used antifungal drugs targeting the ergosterol biosynthetic pathway has been extensively investigated in trypanosomatids. It has been observed that several drugs effective against fungi also demonstrate activity against *Leishmania* spp. and *Trypanosoma cruzi* (Goad et al., 1984; Vannier-Santos et al., 1995; Urbina, 1997; Yao and Wilson, 2016).

Considering the similarities observed between fungi and trypanosomatids and the reports that FOH influences the development of *C. albicans*, the interference of the isoprenoid FOH on the proliferative ability of *Leishmania amazonensis* promastigotes and its presence in the lipid secretome of the parasite were investigated.

## Materials and methods

### Maintenance and cultivation of parasites

*L. amazonensis* promastigotes (strain MHOM/BR/77/LTB0016) were maintained at 26°C in Schneider’s medium (Sigma-Aldrich, St. Louis, USA) supplemented with 5% fetal bovine serum (FBS), 100 μg/ml streptomycin and 100 U/ml penicillin. Alternatively, the parasites were grown in a nutritionally restricted medium with defined chemical composition, characterized by the absence of lipids and FBS. This medium consisted of a mixture of RPMI media (Sigma- Aldrich, St. Louis, USA) and DMEM (Invitrogen Corporation, GIBCO, Leiden, Netherlands) in the proportion of 1:1 (v/v). The composition of the nutritionally restricted medium was adapted from the protocol elaborated by Merlen et al. (Merlen et al., 1999), which has been described as suitable for the cultivation of several strains of *Leishmania* spp. and also of *Trypanosoma cruzi* epimastigotes. The main modification concerning the original protocol consisted of withdrawal of cholesterol supplementation. Regarding the other supplements used, the concentrations were maintained similar to those established by the authors.

### Acquisition and storage of *t, t*-FOH

The isoprenoid *t, t*-FOH (96% purity) was purchased from Sigma-Aldrich. After the bottle was opened, all contents were aliquoted, in the dark environment, for small bottles of amber glass. Immediately before sealing the flasks, the internal atmosphere was saturated with nitrogen gas (N_2_). The aliquots’ vials were stored in a container with silica at low temperatures (-20°C) until use.

### Analysis of *t, t*-FOH activity on the culture growth of *L. amazonensis* promastigotes

To analyze the activity of *t, t*-FOH on the growth of *L. amazonensis*, promastigotes were cultured in Schneider’s medium (5% FBS) or nutritionally restricted medium containing different concentrations of *t, t*-FOH. The initial inoculum was 5x10^5^ parasites/mL, and the concentration of promastigotes in the culture was determined after 24, 48, and 72 h by counting the parasites diluted in formalin (4%) using a hemocytometer. Graphs and IC_50_ values were obtained from the GraphPad Prism software (version 7; GraphPad Software, Inc., La Jolla, CA, USA).

### Evaluation of *t, t*-FOH effects on the cell division of *L. amazonensis* promastigotes

To assess whether *t, t*-FOH interfere with the cell division of *L. amazonensis*, carboxyfluorescein succinimidyl ester (CFSE) labeled promastigotes were cultured in Schneider’s medium or in the nutritionally restricted medium in the presence of *t, t*-FOH. Labeling of the parasites was performed by incubating a suspension of 2.5 x 10^7^ parasites/ml in PBS (0.1% bovine albumin) with 10 μM CFSE (Kit Cell Trace, Molecular Probes/Life Technologies) for 15 minutes at 26° C and protected from light. After this time, the reaction was stopped using cold Schneider’s medium (5% FBS). After labeling, the promastigotes were incubated with 37 µM in Schneider’s medium or 7.5 µM in the nutritionally restricted medium of *t, t*-FOH. The fluorescence intensity of the CFSE was analyzed 24, 48, and 72 h after labeling with CFSE. The data were acquired using a FACSCalibur flow cytometer equipped with the Cell Quest program. The obtained data were analyzed using the Summit v4.3 computer program.

### Evaluation of cell cycle

Promastigotes treated with 46 µM of *t, t*-FOH, corresponding to the IC_50_, were fixed with 70% ethanol, and maintained at -20° C for one hour. Next, the cells were centrifuged, and the pellet was resuspended and incubated for one hour at 26° C in 500μl of RNAse (200 μg/mL) and solubilized in PBS. Subsequently, 20 μL of propidium iodide (PI, 40μg/mL) were added, and the cells were incubated in the dark for 20 minutes at room temperature (Sen et al., 2007). The data were acquired using a FACSCalibur flow cytometer equipped with the Cell Quest program. The data were analyzed using the Summit v4.3 computer program.

### Evaluation of DNA fragmentation

DNA fragmentation in promastigotes was analyzed using a terminal deoxyribonucleotide transferase-mediated dUTP nick-end labeling (TUNEL) apoptosis detection system (Promega, Madison, WI, USA) to the manufacturer’s recommendations. Briefly, 5×10^6^ promastigotes were collected after a 48 h treatment with 46 µM of *t, t*-FOH, washed twice with PBS, and fixed with fixation/permeabilization solution (eBioscience, San Diego, CA, USA) for 10 minutes at 25°C. The fixed cells were incubated with a TdT reaction mixture containing FITC-labelled dUTP for 1 h at 26°C. Cells were washed and resuspended in 0.5 mL of PBS (pH 7.4) containing 0.5 mg/mL PI (BD Biosciences) before analyzing using a FACSCalibur flow cytometer. DNAse was utilized as a positive control.

### Evaluation of mitochondrial membrane potential (ΔΨm)

The mitochondrial functionality of *L. amazonensis* promastigotes were evaluated by flow cytometry using the rhodamine 123 fluorescent probe (Rho 123). In these analyzes, the promastigotes (1x10^6^ parasites/mL) treated with 46 µM of *t, t*-FOH for 24 hours were incubated with 10 μg/mL of Rho 123 (Sigma-Aldrich, St. Louis, USA) for 20 minutes at room temperature and protected from light. In addition, promastigotes incubated under the same conditions with miltefosine (20 μM) were used as a positive control of the assay. The data were acquired using a FACSCalibur flow cytometer equipped with the Cell Quest program. The analysis of the data obtained by the cytometer was performed using the Summit v4.3 computer program.

### Extraction and identification of isoprenoids in secretome of *L. amazonensis* promastigotes

*L. amazonensis* promastigotes were cultivated in the nutritionally restricted medium using glass bottles and subjected to slight agitation. Initially, the promastigotes in the culture were removed by centrifugation (3000 rpm/15 minutes), followed by sterilization by a filtration membrane filter of 0.22 μM pore size (Merck Millipore, Brazil). Next, an aliquot of 0.5 μg of progesterone was added to the sterile supernatant, which was used as the internal control of the extraction technique. For lipid extraction, we used ethyl acetate (99.9% purity, Sigma-Aldrich). Subsequently, the ethyl acetate in the sample was evaporated using a rotary evaporator, and to remove residual solvent, nitrogen gas (N_2_) was used. Finally, the dried samples were stored at -20°C until analysis of the compounds.

Lipids extracted from the secretome were analyzed by chromatography gas and mass spectrometry (GC/MS). The dried samples were resuspended in 100 μL of ethyl acetate immediately before being injected into the equipment GCMS-QP2010 Ultra (Shimadzu Scientific Instruments, Tokyo, Japan). After injection, the column temperature was maintained at 50° C for 1 minute and then increased to 270°C in a ratio of 10° C/min and finally to 300°C in a ratio of 1° C/min. The helium gas flow was kept constant at 1.1 ml/min. The injector and detector temperatures were 250°C and 280°C, respectively (Torres-Santos et al., 2009).

### *In silico* analysis

To perform *in silico* analysis and identify putative farnesene synthase sequences, we gathered annotated sequences of the enzyme. A total of 109 protein sequences from plants and bacteria were obtained from RefSeq (NCBI). Given the need to focus on distant homologs, we emplyed the Hidden Markov Model (HMM profile) approach (Eddy, 1996). Initially, the sequences were aligned using MAFFT 7 software (Katoh and Standley, 2013). Subsequently, we constructed an HMM protein model using hmmbuild from HMMER 3.2.1 (Finn et al., 2011) and utilized hmmsearch to search the profile against the predicted proteins of *Leishmania amazonensis*, obtained from the Laboratory of Computational Biology and Systems. Concurrently, an OrthoMCL analysis (Li et al., 2003) was conducted, involving seven species of the *Leishmania* genus (*L. panamensis, L. infantum, L. braziliensis, L. amazonensis, L. major, L. donovani*, and *L. mexicana*), to identify orthologs within these species. Following this, with the validation of InertPro (Mitchell et al., 2019), we successfully identified putative farnesene synthase protein sequences in each *Leishmania* species.

## Results

### Evaluation of the effect of *t, t*-farnesol on the growth of *L. amazonensis* promastigotes

**Figure 1:**
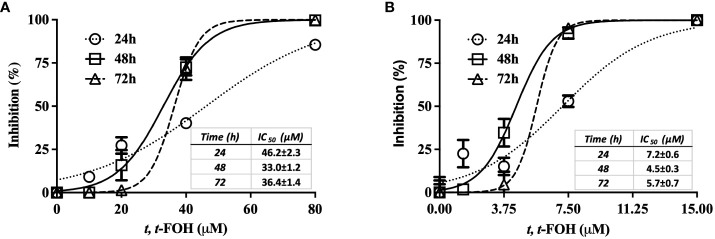
Effect of *t, t*-FOH on the parasite growth. *L. amazonensis* promastigotes were cultured in Schneider´s medium supplemented with 5% FBS (nutritionally rich medium) **(A)** or in a nutritionally restricted medium **(B)** containing different concentrations of *t, t*-farnesol (*t, t*-FOH).

The effect of *t, t*-FOH on *L. amazonensis* promastigotes growth was evaluated using a nutritionally rich medium composed of Schneider´s medium supplemented with 5% FBS (**Figure 1A**) or a nutritionally restricted medium (**Figure 1B**) containing different concentrations of *t, t*-FOH. The results indicate that the concentration of *t, t*-FOH required to inhibit 50% of the growth of *L. amazonensis* is significantly higher in a nutritionally rich medium compared to a nutritionally restricted medium, with the respective IC_50_ values (µM) as follows: 24 h (46.2 ± 2.3 vs 7.2 ± 0.6), 48 h (33.0 ± 1.2 vs 4.5 ± 0.3) and 72 h (36.4 ± 1.4 vs 5.7 ± 0.7).

### Evaluation of *t, t*-FOH effects on cell division of *L. amazonensis* promastigotes

To evaluate the cell division of *L. amazonensis* promastigotes, the parasites were labeled with CFSE and then cultured in a nutritionally rich or restricted medium containing the IC_50_ of *t, t*-FOH, and the fluorescence was measured after 24, 48, and 72 hours.

**Figure 2:**
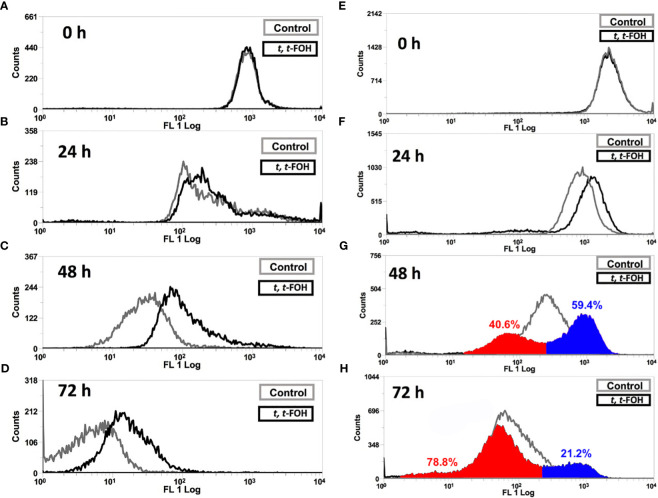
Effect on cell division of *L. amazonensis* promastigotes. Promastigotes of *L. amazonensis* labeled with CFSE were grown in a medium nutritionally rich (Schneider´s medium, 5% FBS) **(A-D)** containing 37 µM or nutritionally restricted medium **(E-H)** containing 7.5 µM of *t, t*-FOH. After 24, 48, and 72 h, the proliferative capacity of the parasites was analyzed using an FACSCalibur flow cytometer.

**Figures 2A-D** show the cell division of *L. amazonensis* promastigotes grown in a nutritionally rich medium containing 37 μM of *t, t*-FOH. By analyzing the histogram obtained after 48 hours of incubation, it is observed that there was a decrease in the number of cell divisions of the parasite.

**Figures 2E-H** show the cell division profile of the parasites grown in a nutritionally restricted medium with 7.5 μM of *t, t*-FOH. Interestingly, *t, t*-FOH had a very distinct effect on cell division of the promastigotes in the poor medium. We can observe that the parasites are distributed in two different populations, with a particular pattern of cell division. One group divides faster than the untreated control, while the other divides slower. It is more evident in 48 hours of culture when 40.6% of the treated cells divided faster than the median of the untreated parasites, while the remaining 59.4% divided slower. This is partially reversed in 72 h, possibly because the faster group reached the stationary phase, and the control continued to divide.

### Evaluation of *t, t*-FOH effects on the cell cycle of *L. amazonensis* promastigotes

**Figure 3:**
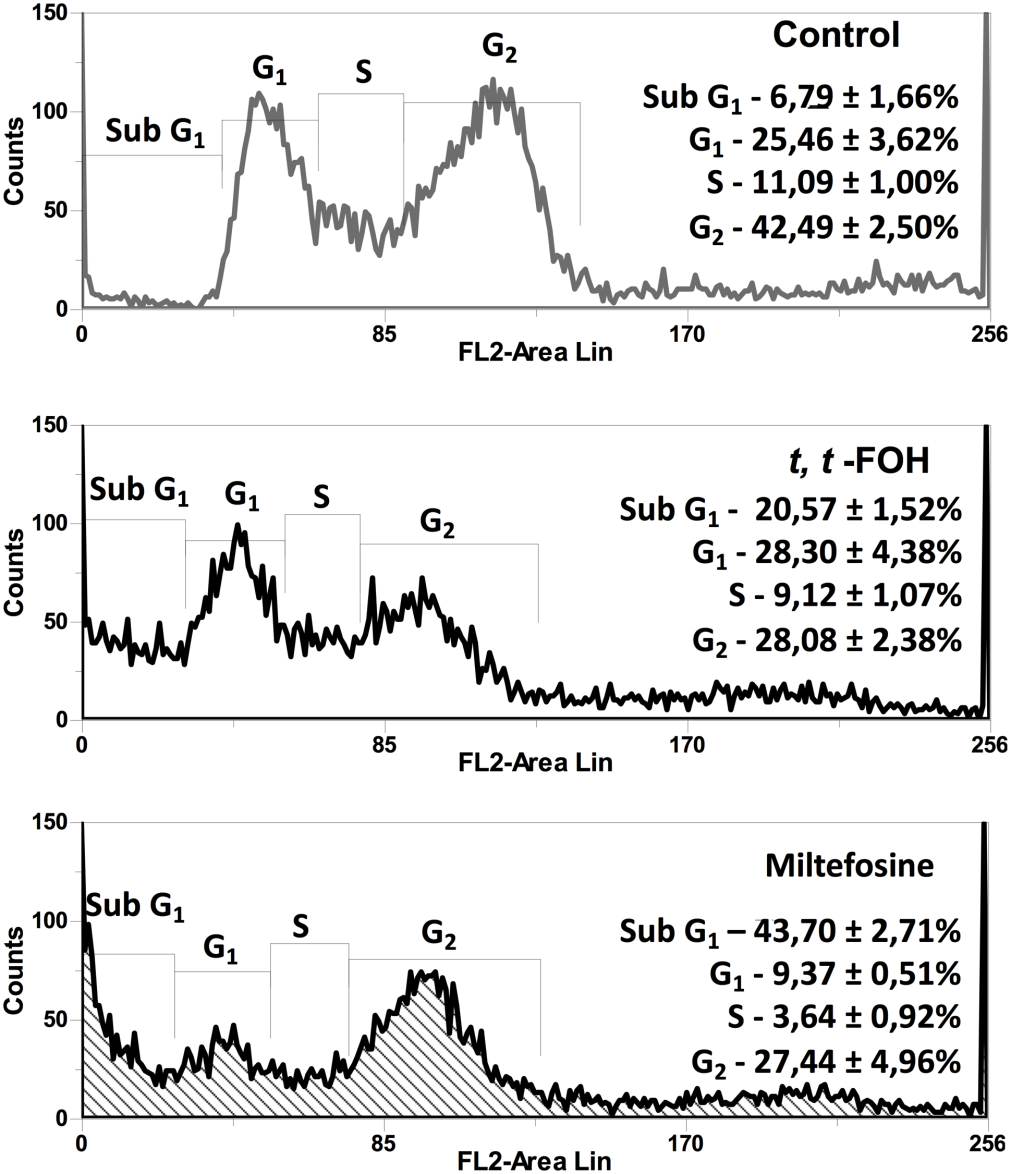
Effect of *t, t*-FOH on the cell cycle on *L. amazonensis* promastigotes. The parasites were grown in a nutritionally rich medium (Schneider, 5% FBS) containing IC_50_ of *t, t*-FOH, or 20 μM miltefosine. After 24h, a culture sample was labeled with propidium iodide (PI) and analyzed using a FACSCalibur flow cytometer. The percentage of cells in each cell cycle phase is described in the histograms.

In this assay, we evaluated whether *t, t*-FOH reduces the proliferative capacity of the promastigotes by interfering in the cell cycle of the parasite. The promastigotes were cultured in the nutritionally rich medium with the concentration of *t, t*-FOH corresponding to the IC_50_ (46 µM). After 24 hours, a culture sample was labeled with PI and analyzed by flow cytometry. **Figure 3** shows that after the promastigotes remained incubated with the IC_50_ value of *t, t*-FOH, there was a significant decrease in cells in the G_2_ phase, compatible with a stop in the cell cycle. At the same time, we also observed an increase in cells in the region corresponding to Sub-G1, that is, with less DNA than G1. The occurrence of these hypodiploid cells may be suggestive of apoptosis.

### Evaluation of *t, t*-FOH effects on DNA fragmentation in promastigotes of *L. amazonensis*

**Figure 4:**
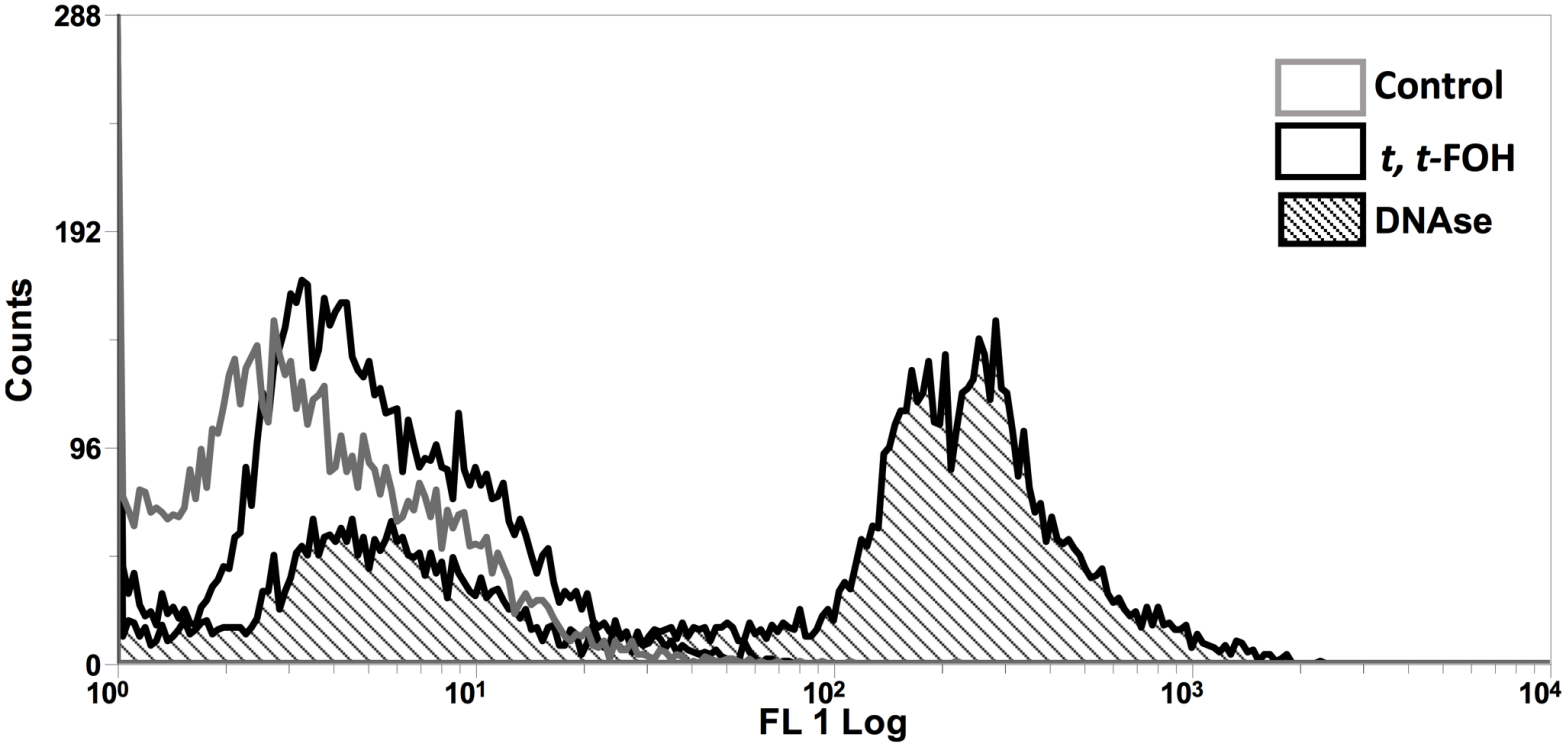
Effect of *t, t* -farnesol on DNA fragmentation in promastigotes of *L. amazonensis*. Promastigotes were incubated with IC_50_ or DNAse for 48 h, and then a TUNEL assay was performed. The fluorescence was analyzed by flow cytometry. DNAse was utilized as a positive control. The histogram is representative of three independent experiments.

To investigate the increase of promastigotes in the sub-G_0_/G_1_ phase after incubation with *t, t*-FOH, we looked for DNA fragmentation in the parasites. Analysis of the effect of *t, t*-FOH on the promastigotes cultured in a nutritionally rich medium was done after 48 hours of incubation, and the concentration of *t, t*-FOH used was equal to the IC_50_ (46 µM). However, despite the hypodiploidy observed in the cell cycle analysis, there was no DNA fragmentation in parasites incubated with *t, t*-FOH (**Figure 4**).

### Determination of *t,t*-FOH effects on mitochondrial membrane potential (ΔΨ_m_) of L. amazonensis promastigotes

**Figure 5:**
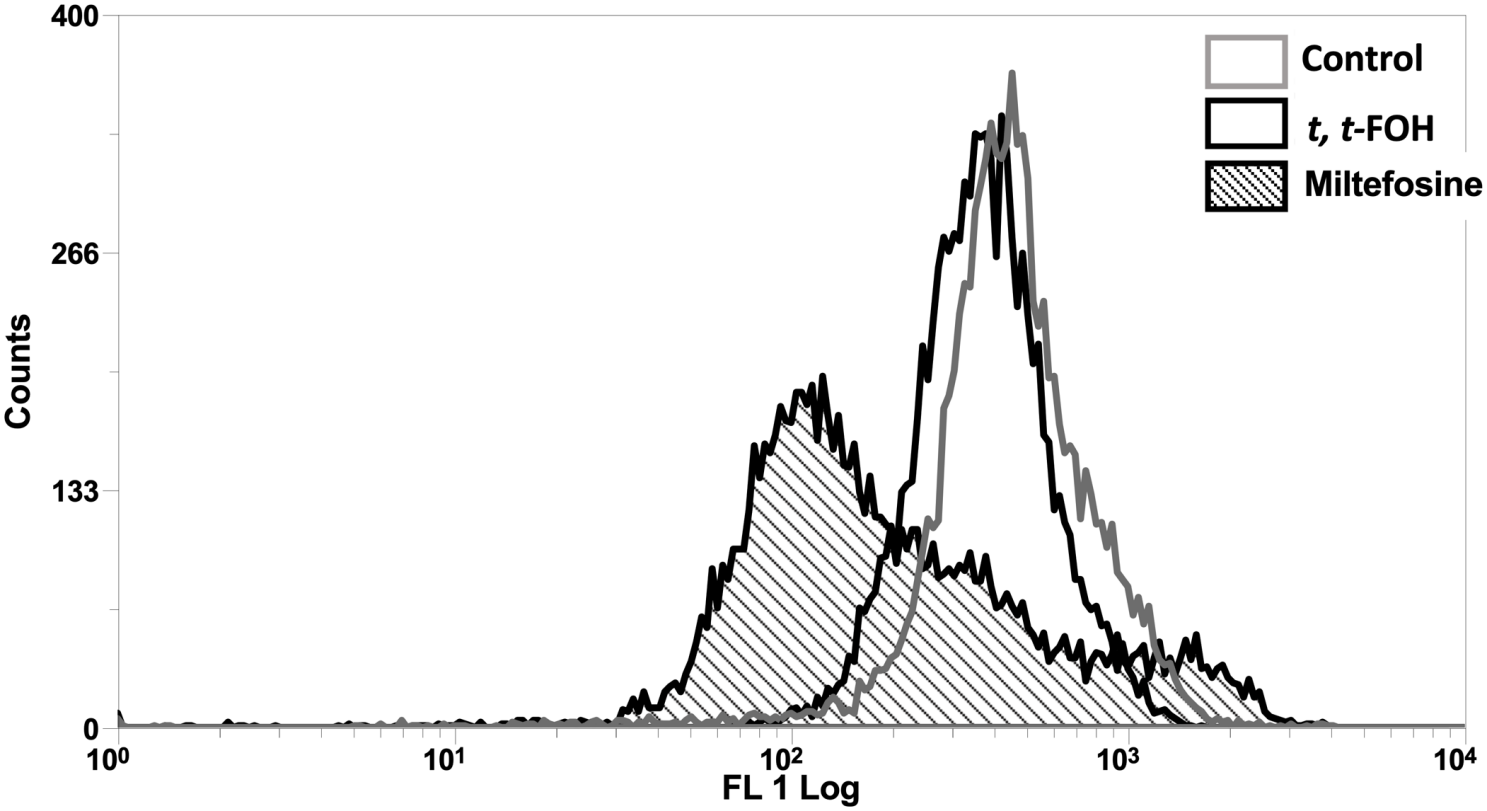
Effect of *t, t* FOH on the mitochondrial membrane potential (ΔΨ_m_) of promastigotes of *L. amazonensis* grown in a nutritionally rich medium. Promastigotes of *L. amazonensis* were cultured in a schneider medium (5% of PBS) containing IC_50_ *t, t*-FOH. After 24h, the mitochondrial membrane potential (ΔΨ_m_) was analyzed by flow cytometry using Rhodamine 123 (Rho123). Miltefosine was used as a positive control of the test. The histogram is representative of three independent experiments.

Mitochondrial membrane potential (ΔΨ_m_), another hallmark of apoptosis, was also investigated in cells incubated with *t, t*-FOH. Promastigotes cultured in a nutritionally rich medium (Schneider´s medium supplemented with 5% FBS) were treated with *t, t*-FOH corresponding to the IC_50_ (46 µM). After 24 hours, the ΔΨ_m_ was assessed by flow cytometry. **Figure 5** shows that *t, t*-FOH does not interfere with the mitochondrial membrane potential of the parasites.

### Identification of isoprenoid derivatives in the secretome of promastigotes of *L. amazonensis*

To extract lipophilic metabolites in the culture supernatant, promastigotes of *L. amazonensis* were cultivated for ten days. The lipids were extracted on days 1, 4, 7, and 10 of the culture supernatants. The days 1 and 4 represent the logarithmic phase, and the days 7 and 10 illustrate the stationary phase of the cultures.

**Figure 6:**
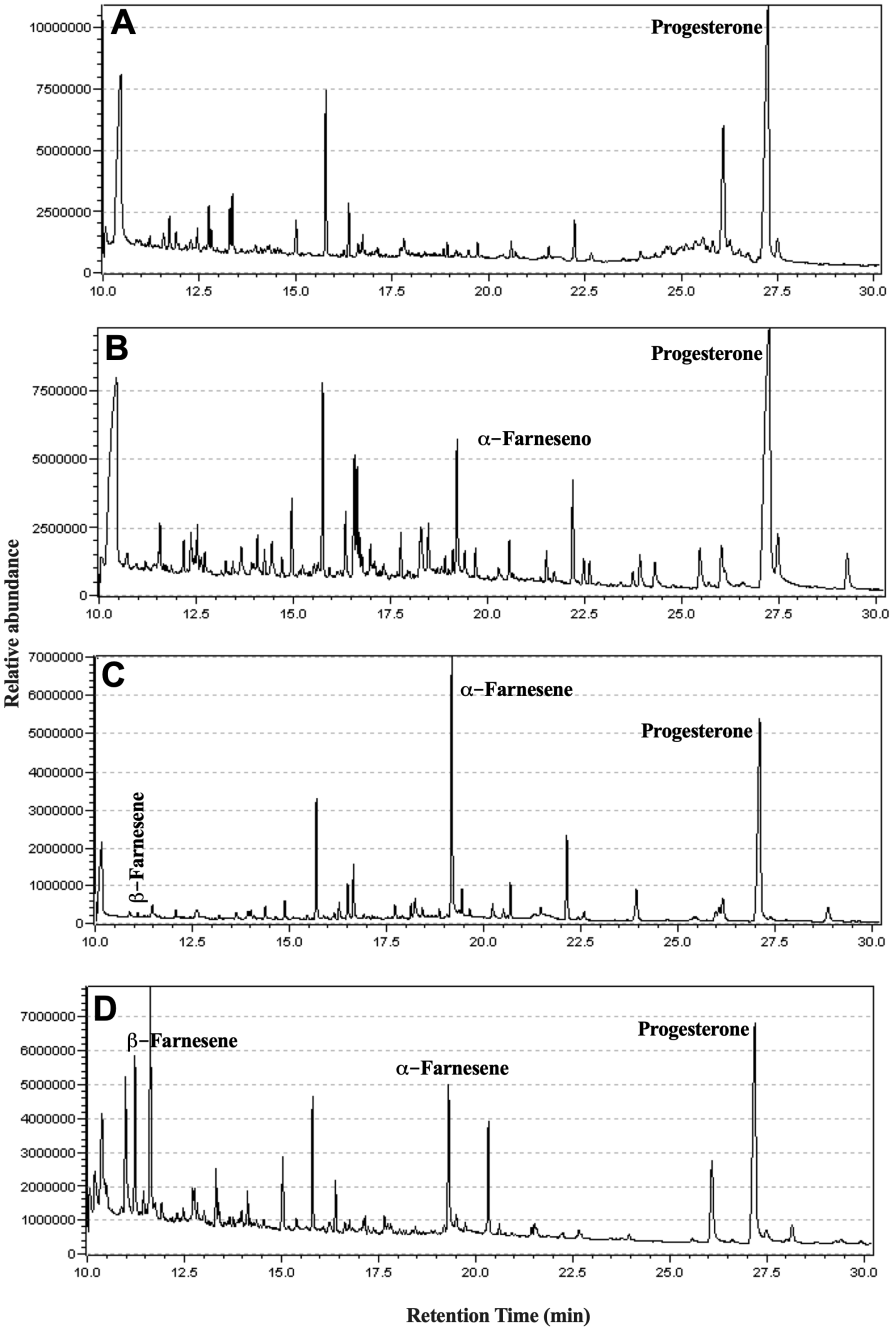
Identification of isoprenoids in the culture supernatant of promastigotes of *L. amazonensis*. Promastigotes were cultured in medium culture nutritionally restricted in the absence of lipids and fetal bovine serum. On days 1 **(A)**, 4 **(B)**, 7 **(C)**, and 10 **(D)**, the lipophilic metabolites present in the supernatant were extracted using ethyl acetate and identified by GC-MS. Data representative of a total of two experiments.

GC-MS analysis was conducted to examine the neutral lipids in the supernatant, and the representative chromatograms are depicted in **Figure 6**. The compound β-farnesene was detected starting from day four, while α-farnesene was observed in increasing concentrations on days 7 and 10 (**Table 1**).

**Table 1:** Identification of isoprenoids in the culture supernatant of promastigote of *L. amazonensis*.

Compound	MW	Relative amount (%) *			
		D1	D4	D7	D10
α−**Farnesene**	204.357	–	11.53	37.13	19.43
β−**Farnesene**	204.357	-	-	0.9	19.03

* The relative abundance was calculated using progesterone (internal standard). -, not detected.

**Figure 7:**
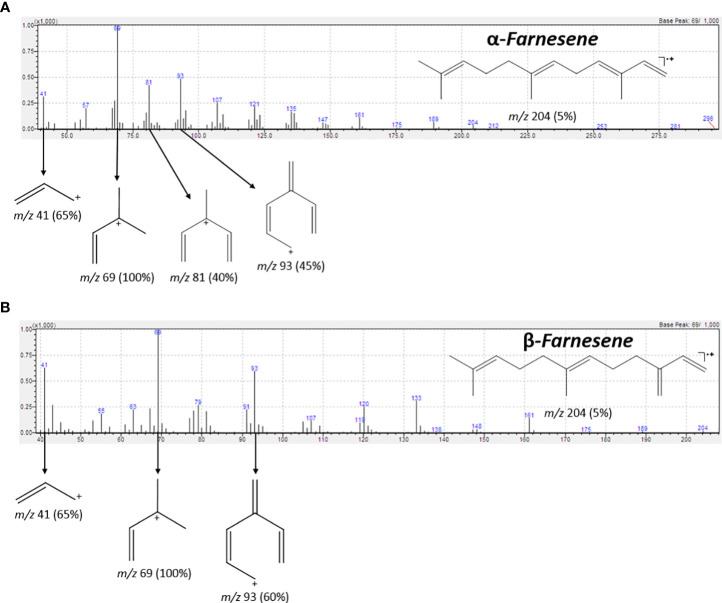
IE-MS spectrum and the fragmentation pathway of α-farnesene **(A)** and β-farnesene **(B)**. Highlight for the fragment m/z 81 for **(A, B)**. The difference in intensity of peaks m/z 81 in **(A, B)** can be explained in terms of the stability of the carbocations formed, and the ring stresses in the structures in **(B)**.

The analysis of the chromatograms and mass spectra obtained by GC-MS, with assistance from the NIST11 spectral library, revealed the presence of both α-farnesene and β-farnesene isomers. Although the spectra are highly similar, a notable distinction lies in the intensity of the peak at *m/z* 81, which constitutes 50% of the total intensity in the mass spectrum of α-farnesene. This distinction can be explained through the proposed fragmentation mechanisms, which indicate potential differences in the stability of the *m/z* 81 fragments between the two isomers (**Figure 7**). Specifically, the fragmentation of β-farnesene, through sigma cleavage between the C5-C6 carbons, leads to a fragment at *m/z* 81 with resonance structures alternating between a primary and a secondary carbocation. In contrast, the same fragmentation mechanism for α-farnesene results in resonance structures alternating between a primary and a tertiary carbocation, with the latter offering greater stability to this fragment. This accounts for higher intensity of the *m/z* 81 peaks in the mass spectrum of α-farnesene. Another contributing factor to the lower stability of the *m/z* 81 fragments obtained from β-farnesene is the ring tension within the proposed fragment structure.

### Identification of farnesene synthase in *Leishmania* spp.

**Figure 8:**
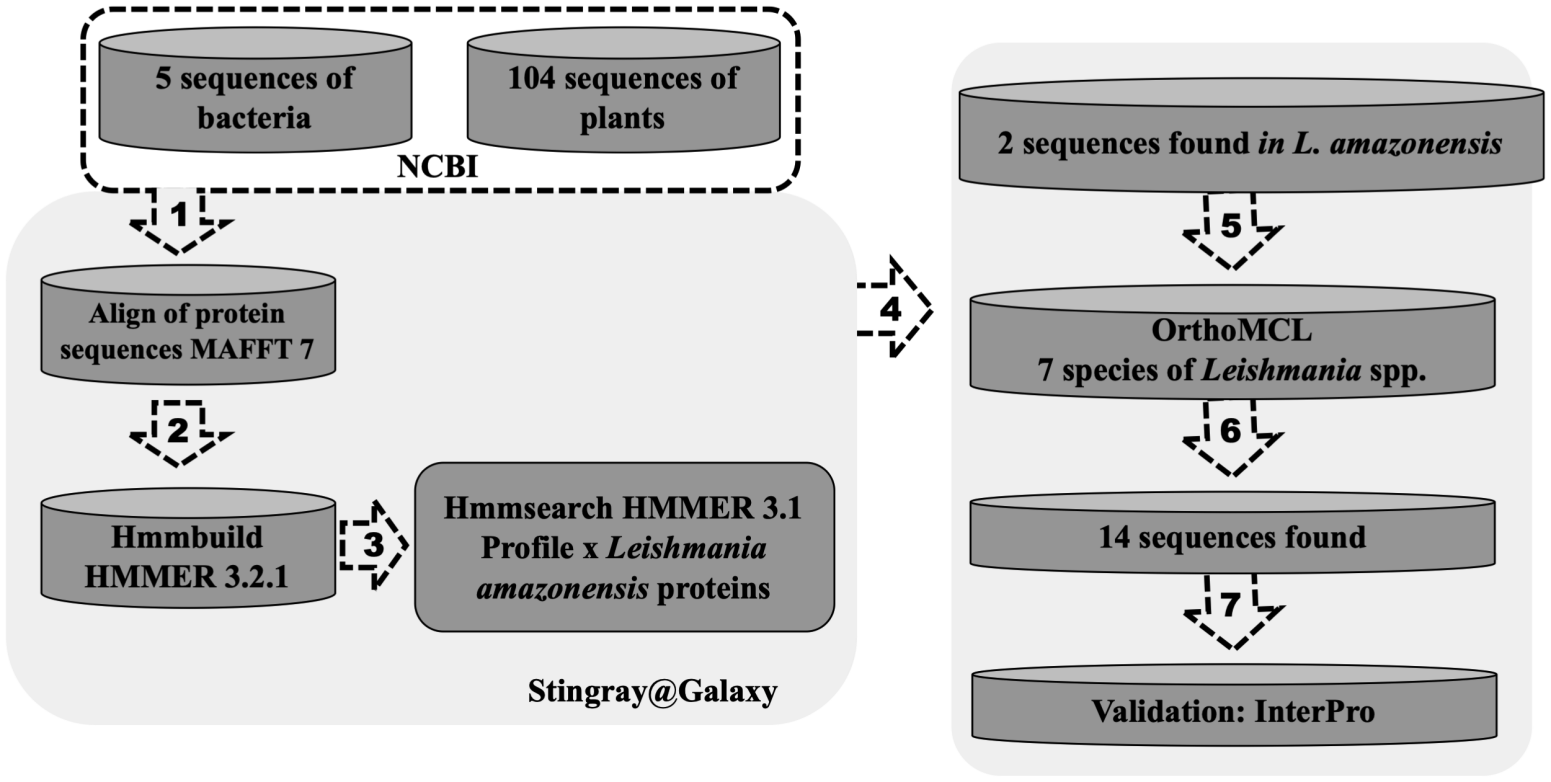
*In silico* prospection of a farnesene synthase putative protein sequence in *Leishmania* spp. One hundred four protein sequences of farnesene synthase from plants and bacteria were obtained from RefSeq (NCBI) and (1) aligned using software MAFFT. After that, (2) the HMM profile was built with hmmbuild from HMMER 3.2.1, and then using (3) hmmsearch to search the profile against the predicted proteins of *Leishmania infantum* obtained from TriTrypDB. Using this approach, two sequences were obtained in *L. infantum*. Meanwhile (4), an OrthoMCL analysis was performed using seven species of the *Leishmania* genus (*L. panamensis, L. infantum, L. brazilensis, L. amazonensis, L. major, L. donovani* and *L. mexicana*) to identify orthologs of these species. After that, it was possible to (5) find the orthologs groups of these two sequences found in *L. infantum* (6), totalizing 14 sequences from 7 species separated into two orthologs groups. After the validation using InertPro (7), it was possible to identify a farnesene synthase putative protein sequence in each species of *Leishmania*.

α- and β-farnesene were found in the secretome of *L. amazonensis*, prompting an investigation into the enzyme responsible for this reaction, which is interconnected with the mevalonate pathway. This enzyme, previously described in plants (Pechous and Whitaker, 2004) and annotated in RefSeq-NCBI as farnesene synthase, was sought after. Using these sequences, we initiated a search for distant homologs to identify a corresponding sequence in *Leishmania* spp. Through this *in silico* analysis, we identified a putative farnesene synthase for *Leishmania* spp. (**Figure 8**). This search allowed us to identify a putative homologous sequence of farnesene synthase in *L. amazonensis*, based on a profile constructed using previously annotated sequences from different species. Utilizing the OrthoMCL software, which included seven other species of *Leishmania*, including *L. amazonensis*, we determined the homologous group to which it belonged based on its sequence identifier. Subsequently, we verified the classification of the enzymes in *Leishmania* and the bacteria used to construct the HMM profile with InterPro, aiming to validate the putative homolog. In this *in silico* validation, we found that all sequences exhibited similar characteristics to the farnesene synthase found in bacteria. These characteristics include protein family membership, involvement in the same biological processes, and molecular functions (**Table 2**).

**Table 2:** Identification of farnesene synthase putative protein sequence in each species of *Leishmania*.

Specie	Sequence ID	Database	InterPro
*L. infantum*	LinJ.34.3110:mRNA	TriTryp	Cytochrome P450, E-class, group I (^*^^1^IPR002401) Biological Process ^*^^2^GO:0055114 oxidation-reduction process Molecular Function ^*^^2^GO:0005506 iron ion binding ^*^^2^GO:0016705 oxidoreductase activity, acting on paired donors, with incorporation or reduction of molecular oxygen ^*^^2^GO:0020037 heme binding
*L. panamensis*	XP_010698508.1	RefSeq	
*L. amazonensis*	LAJMNGS018C06.b.3162	^*^^3^LBCS	
*L. braziliensis*	LbrM.20.2920:mRNA	TriTryp	
*L. mexicana*	LmxM.33.3330.1	TriTryp	
*L. major*	LmjF.34.3330:mRNA	TriTryp	
*L. donovani*	LdBPK_343110.1.1	TriTryp	
*Planktotalea frisia*	OJI93009.1	RefSeq	

^*^^1^ IPR InterPro identifier ^*^^2^ GO Gene Onthology identifier ^*^^3^ Laboratory of Computational Biology and Systems

## Discussion

The first step of the work consisted in evaluating the effects of *t, t*-FOH on *L. amazonensis* growth. We observed a different pattern between parasites grown in the rich or poor medium in the presence of *t, t*-FOH. A higher FOH concentration was required to inhibit the growth of the parasites in the rich medium (**Figure 1**). Similar results were also described by Langford et al. (2010). The authors analyzed the culture conditions in that FOH promotes toxicity or differentiation in *C. albicans*. They explained that cultures of *C. albicans* maintained in a nutritionally rich environment were more tolerant to the treatment with FOH in comparison to the cells maintained in PBS. From this observation, the authors suggested that the mechanism of tolerance of *C. albicans* to FOH consists of physiological adaptation depending on energy availability. Thus, the richer in nutrients is the culture medium in which the fungus grows, the proportionally higher will also be the cell’s ability to tolerate higher concentrations of FOH in the medium. Another aspect that has also been described favoring the mechanism of tolerance of *C. albicans* to FOH consists of the albumin present in the FBS. Mosel et al. suggested that serum albumin can bind to FOH, which would result in FOH blockade in the culture and consequently in greater tolerance of the fungus to the compound (Mosel et al., 2005).

We then evaluated the effect of *t, t*-FOH on the cell division of *L. amazonensis* promastigotes cultured in nutritionally rich or restricted medium containing the IC_50_ of *t, t*-FOH (**Figure 2**). The results showed that *t, t*-FOH decreased the number of cell divisions of *L. amazonensis* promastigotes grown in rich medium, mainly after 48 e 72 hours of incubation, suggesting a delay in cell cycle. Intriguingly, the impact of *t, t*-FOH in the nutrient-poor medium exhibited a dual effect. Some cells divided faster than the control, while others divided more slowly. This outcome suggests the presence of subpopulations of promastigotes that respond differently to *t, t*-FOH under nutritional stress. It’s important to note that although we often treat promastigotes as a homogeneous population, they are known to differentiate into at least four distinct developmental stages inside the sand fly midgut or in culture (procyclic, nectomonad, leptomonad, and metacyclic), influenced by nutrient availability and other environmental factors. Recent work by Coutinho-Abreu and colleagues demonstrated that these forms, when directly collected from the invertebrate host, exhibit differential gene expression (Coutinho-Abreu et al., 2020). As the first three forms are replicative, while the infective metacyclic form is not, it is possible that *t, t*-FOH serves as a signal for the proliferation of a specific differentiation stage under nutritional stress. Over time, this population may reach a stationary phase and transform into non-replicative metacyclic promastigotes, as suggested by the data in 72 h (**Figure 2H**). Further experiments are needed to investigate this hypothesis.

Then, we assessed whether *t, t*-FOH reduces proliferative ability by interfering with the cell cycle of the parasite (**Figure 3**). After the promastigotes remained incubated for 24 hours with the IC_50_ of *t, t*-FOH occurred a significant decrease in the percentage of cells in the G_2_ phase and an increase of cells in sub G_0_/G_1_ phase. However, no significant difference was observed in the percentage of promastigotes distributed in the other cell cycle stages (G1 and S phases). These results show that the growth inhibition seen after treatment with FOH is partly due to a reduction in the number of cells cycling. Furthermore, some toxicity was also observed once a hypodiploid population was noticed.

To evaluate whether the observed toxicity was due to apoptosis, we analyzed the action of the isoprenoid on DNA integrity and the mitochondrial functionality of the parasites (**Figures 4**, **5**). Interestingly, the promastigotes treated with the IC_50_ of *t, t*-FOH showed preserved mitochondrial membrane potential and DNA integrity. The functionality of mitochondria is an important indicator of cell viability since this organelle is the main metabolic energy-generating center. So, the permeabilization of the mitochondrial membrane and consequent dysfunction of the organelle is often a decisive event to determine cell death (Kroemer et al., 2007). These results showed that the parasites were not in the process of apoptosis after 48 hours of incubation with *t, t*-FOH, considering that loss of mitochondrial membrane potential and DNA fragmentation are two of the main characteristics of this kind of death (Henry et al., 2013).

A possible explanation for the emergence of the hypodiploid population in the treated culture could be the performance of *t, t*-FOH as a detergent, causing the death of a small number of parasites due to necrosis. Indeed, it has already been demonstrated in bacterial and fungal cultures that the hydrophobic nature of FOH promotes its action as a detergent due to accumulation in membranes, resulting in cell rupture immediately afterward (Shirtliff et al., 2009).

After studying the effects of *t, t*-FOH on cultures of *L. amazonensis* promastigotes, we analyzed the lipophilic content in the secretome of *L. amazonensis* (**Figure 6**). To guarantee that the parasite released the lipids present in the culture supernatant, we used a nutritionally restricted culture medium whose composition is known and characterized by the absence of lipids and FBS. Thus, the possibility that the lipids identified were from an exogenous source of FBS was discarded. Some authors have also used similar strategies to attend to the need to grow parasites without FBS because it could interfere with the results of studies involving biochemical and immunological analyzes, due to the lack of knowledge of the exact composition of the product, besides the heterogeneity from batch to batch that would reflect in variation in the results (Merlen et al., 1999; Santarém et al., 2014).

The search for isoprenoid derivatives in the secretome of *L. amazonensis* resulted in identifying α-farnesene and β-farnesene in the culture (**Table 1**) and *in silico* analysis indicated that the parasite has the DNA sequence for the farnesene synthase enzyme. The time interval between days 7 and 10 is representative of the stationary phase of the growth curve of the culture. It has already been shown that isoprenoids can perform signaling activity in cultures of *C. albicans* (Shchepin et al., 2003).

Interestingly, a set of sesquiterpenoids described in the literature are referred to as juvenile hormones (HJs). Juvenile hormones have been extensively studied because of their importance to the physiology of insects, playing central roles in the embryonic development, metamorphosis, and reproduction of these arthropods. Among the HJs, juvenile hormone III (HJ III) is the most commonly expressed among insects (Sperry and Sen, 2001; Cao et al., 2009). The mevalonate biosynthetic pathway synthesizes HJ III, and FOH production is critical for the endogenous control of the synthesis of this hormone (Cao et al., 2009). In addition, it has been reported that the presence of precursors is the limiting factor for the production of HJs (Bloch et al., 2013). According to these data, the presence of FOH is fundamental for the flow of isoprenoids along pathways that are compromised with the production of these hormones.

Thus, the data in the literature describing sesquiterpenes as essential molecules for insect physiology and the identification of isoprenoid derivatives in the culture supernatant of *L. amazonensis* promastigotes suggest that these compounds can act as members of some type of interaction established between the parasite and its invertebrate host. *Leishmania* and sand flies constitute one of the oldest pairs of parasite-vector. Throughout this co-evolutionary process, the parasite developed varied adaptive mechanisms to guarantee success in establishing infection (Bates, 2008; Ramalho-Ortigao et al., 2010). It has been described that trypanosomatids have a secretory system adapted to the parasitic lifestyle (McConville et al., 2002). *Leishmania* secretes compounds such as chitinases, neuropeptides, and different types of glycoconjugates. All these products are supposed to act to affect the phlebotomine physiology or behavior to guarantee the establishment of the infection and the continuation of the cycle with the parasite for the vertebrate host (Kamhawi, 2006). Intriguingly, it has been reported that β-farnesene serves as a feeding stimulant for *Lutzomyia longipalpis* (Tesh et al., 1992). If the promastigotes secrete this isoprenoid in the insect midgut, as observed in culture in this study, the physiological effects are yet to be elucidated. As mentioned earlier, *t, t*-FOH is involved in the biosynthesis of molecules that alert the physiology of sand flies. However, further studies are needed to state that *L. amazonensis* promastigotes secrete some of these sesquiterpenes to interact with its invertebrate host.

In conclusion, *trans*, *trans*-farnesol added exogenously interferes in the proliferation of promastigotes and inhibits the cell cycle without causing DNA fragmentation or loss of mitochondrial functionality. Subsequently, the lipid secretome was analyzed, and α-farnesene and β-farnesene isomers were detected starting on the fourth day of culture, increasing until the tenth. Together, these results demonstrate for the first time the biological activity of farnesol on *L. amazonensis* and the identification of α-farnesene and β-farnesene in the lipid secretome of the parasite.

## Data availability statement

The raw data supporting the conclusions of this article will be made available by the authors, without undue reservation.

## Ethics statement

This study was performed following the Guide for the Care and Use of Laboratory Animals of the Brazilian National Council of Animal Experimentation (COBEA) and had the approval of the Animal Ethics Committee of Oswaldo Cruz Foundation (license number L-02/2022).

## Author contributions

Conceptualization, ET-S; Data curation, VA-N, MB, RM-B, and EC-J; Formal analysis, VA-N, RM-B, and EC-J; Funding acquisition, ET-S; Investigation, LP, VA-N, and EC-J; Methodology, LP, VA-N, MB, and RM-B; Supervision, ET-S; Writing – original draft, LP; Writing – review and editing, ET-S. All authors contributed to the article and approved the submitted version.
